# Blood pressure in preterm infants with bronchopulmonary dysplasia in the first three months of life

**DOI:** 10.1007/s00467-024-06354-0

**Published:** 2024-03-27

**Authors:** Judit Klara Kiss, Anna Gajda, Judit Mari, Csaba Bereczki

**Affiliations:** Department of Paediatrics, University of Szeged, Korányi fasor 14, Szeged, 6720 Hungary

**Keywords:** Neonatal hypertension, Blood pressure, Bronchopulmonary dysplasia, BPD, Chronic lung disease, Acute kidney injury

## Abstract

**Background:**

Neonatal hypertension is common in preterm infants with bronchopulmonary dysplasia (BPD). Our study aimed to examine blood pressure variation in the first three months of life in preterm BPD patients.

**Methods:**

We conducted a retrospective, single-centre study at the Neonatal Intensive Care Unit of the University of Szeged, Hungary. We collected blood pressure data from 26 preterm infants (born at < 30 weeks gestation) with moderate or severe BPD over three years (2019–2021). We calculated the BPD group's daily average blood pressure values and used previously defined normal blood pressure values from a preterm patient group born at < 30 weeks gestation as a reference. We used 19,481 systolic, diastolic and mean blood pressure measurement data separately to calculate daily average blood pressures.

**Results:**

We found a statistically significant correlation between the blood pressure values of the BPD patient group and the reference data. The difference between the blood pressure curve of the group with BPD and that of the reference group was also statistically significant. We also analysed individual patients' daily average blood pressure values and found that 11 patients (42%) had hypertensive blood pressure values for three or more days within the first 90 days of life. Within this group, our statistical analysis showed a 25% chance of acute kidney injury.

**Conclusion:**

The blood pressure of the BPD group not only correlated with but also significantly differed from the reference data. Hypertension lasting three or more days occurred more frequently in patients with acute kidney injury accompanied by BPD.

**Graphical abstract:**

**Figure:**
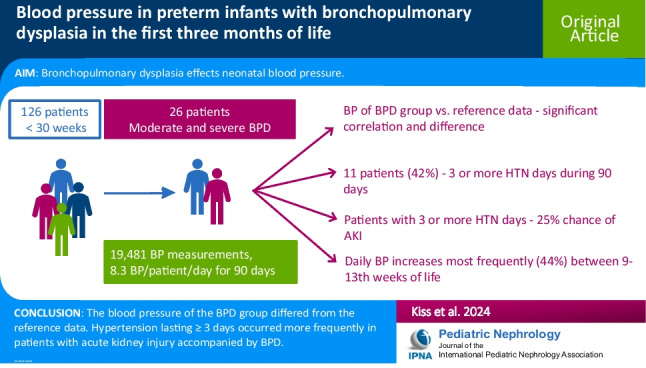
A higher resolution version of the Graphical abstract is available as Supplementary information

**Supplementary Information:**

The online version contains supplementary material available at 10.1007/s00467-024-06354-0.

## Introduction

Neonates treated in neonatal intensive care units (NICUs) have an incidence of hypertension between 1% and 2.5% [1, 2]. Previous research has shown that preterm infants with bronchopulmonary dysplasia (BPD) have a significantly higher risk of developing systemic hypertension [1–8]. According to the literature, the incidence of hypertension varies widely, with rates between 12 and 43% in preterm infants with BPD, also known as chronic lung disease of infancy [3, 6]. Despite improved neonatal care, BPD remains one of the most common complications of preterm birth, affecting 17–75% of preterm infants born before the 28th week of gestation worldwide [9, 10]. BPD and consequent hypertension can significantly affect premature infants' short- and long-term life outcomes. Despite several studies on this topic, there are unanswered questions regarding the blood pressure changes in premature infants with BPD [3–8, 11–14]. Among these questions, the most important ones are the onset and frequency of hypertensive periods in BPD patients. Previous studies applied different definitions for hypertension and BPD and took less frequent blood pressure measurements or used relatively short continuous time intervals in their investigations. These differences make it difficult to interpret and utilise their results in patient care today. Considering these reasons, our study aimed to investigate the changes in blood pressure in the premature BPD patient group during the first three months of life. The next question of the study was to investigate the number of hypertensive periods and other blood pressure characteristics in individual patients. We used recent definitions of hypertension and BPD and utilised a large amount of measured data and a relatively long interval. This kind of investigation may be the first step towards a systematic analysis of this phenomenon.

## Methods

We conducted a retrospective single-centre study at the Neonatal Intensive Care Unit of the University of Szeged, Hungary, to determine the risk of developing hypertension during the first three months of life in patients with moderate and severe BPD. We collected all blood pressure readings in the first 90 days of life of all preterm infants (born before 30 weeks gestation) with moderate or severe BPD from 1 January 2019 to 31 December 2021. Patients who were transferred out or died before 36 weeks of postmenstrual age (PMA) were excluded from the study. First, we examined the blood pressure data of the entire BPD group. We compared our results to previously defined normal daily average and 95th-percentile blood pressure values from a preterm patient group born at < 30 weeks gestation, which served as a reference [15]. From the reference group, we excluded all the patients with hypertension risk based on the categories of neonatal hypertension described by Flynn [16]. Since we found a difference between the daily average blood pressure curves of the two groups, to understand and explain this phenomenon, we examined the development of the patient's blood pressure individually on a daily and weekly basis.

## Definitions

We independently reviewed and collected data on each patient's respiratory status and corresponding support, established a diagnosis based on the results and used the National Institute of Child Health and Human Development (NICHD) 2001 definition to diagnose moderate and severe BPD [17, 18]. Based on the NICHD criteria, we defined moderate BPD as an oxygen requirement less than 30%, while severe BPD was defined as an oxygen requirement of 30% or more and/or a positive pressure ventilation requirement at 36 weeks PMA. All patients in the studied population also met the criteria for Grade 1–3 BPD based on the NICHD 2019 study, which extended the definition to include the mode of respiratory support [19].

Neonatal hypertension was defined as persistent systolic and/or diastolic measurements above the 95th percentile for PMA [16, 20]. A persistent blood pressure increase was defined as a high blood pressure above the 95th percentile for three or more days. Hypertensive periods lasting less than three days are more likely to be the result of a therapeutic intervention than more extended periods. Therefore, our study examined two groups (infants with less than three hypertensive days and infants with three or more hypertensive days) separately.

We used oscillometric measurements exclusively, except for the individual blood pressure analysis of three patients who had only intraarterial data for the first seven days of life. Blood pressure was measured using an oscillometric device (GE Dash 3000 multiparameter monitor system with the GE DINAMAP blood pressure algorithm) with an appropriately-sized cuff (cuff width-to-arm circumference ratio closest to 0.50) [21, 22]. Blood pressure was measured on the right arm while the patient was in a quiet, awake state or sleeping, according to the unit's guidelines. In cases of contraindication (i.e., cannulation or injury of the right arm), left arm or calf measurements were taken.

We used the modified Kidney Disease: Improving Global Outcomes (KDIGO) definition to diagnose neonatal acute kidney injury (AKI) [23]. According to the definition, both serum creatinine levels and urine output were checked to determine AKI.

## Data collection and handling

Blood pressure values, patient demographics, and medical data were retrieved from Phillips's IntelliSpace Critical Care and Anaesthesia (ICCA) electronic medical records. The ICCA hospital administration system automatically stores the monitoring data in databases. All measured blood pressure raw data values in the records of the ICCA database were retrieved using SQL queries and output was saved as CSV text files. After a data validity check, the text file with the data was transferred to a local SQL database for further analysis. We performed all the main calculations based on the measured numeric blood pressure values and relevant additional raw data in our database with a previously developed standalone software program, PDAnalyzer.

The collected data included all blood pressure records, demographic data, antenatal and postnatal steroids, diuretics, blood transfusions, fluid boluses, inotropic support, ibuprofen administration, and the number of invasive and noninvasive ventilation days. We calculated the daily and weekly average blood pressure values for all patients in the first 90 days of life. We used previously determined 95th-percentile blood pressure values and trendlines as a reference for diagnosing hypertension. These data were obtained from a patient group (gestational age less than 30 weeks) without moderate or severe BPD or other risk factors for hypertension [15]. By linear regression analysis, we created the reference 95th-percentile trendlines at both weekly and daily resolutions. Our reference group's 95th percentile curve indicated a statistically strong significant correlation (R = 0.785, two-sided *p* = 0.012) with the corresponding data previously presented by Dionne [24].

## Statistics

In most cases, the statistical analysis was carried out using the IBM SPSS Statistics program (version 29) and Microsoft Excel Data Analysis module. We used a paired samples t-test to compare paired samples with continuous variables, and in the case of discrete variables, we used a simple binomial test. For verification, the significance level was usually set at *p* < 0.05. The graphs were created with Microsoft Excel version 2009.

## Results

### Patient group characteristics

Over three years, our unit admitted 126 patients born at less than 30 weeks gestation, 26 of whom had moderate to severe BPD. Table 1 lists the demographic characteristics and the general therapies applied to the BPD patient group. Patients were not given antihypertensive medications other than those listed in the table.

**Table 1:** Characteristics of moderate to severe BPD patients and BPD patients with three or more or less than three hypertensive days

	BPD patient group	≥ 3 days with HTN	< 3 days with HTN
Patient number, *n*(%)	26 (100)	11 (42)	15 (58)
Mean GA (range)	26.1 (23–29)	26.2 (23–29)	26.1 (24–29)
Birth weight, (g)	696 (300–1390)	685 (300–1390)	705 (350–1360)
Male/female, *n*(%)	14/12 (54/46)	5/6	9/6
Severe BPD, *n*(%)	12 (46.2)	6 (54.5)	6 (40)
Postnatal steroids, *n*(%)	16 (61.5)	7 (63.6)	9 (60)
Diuretics, *n*(%)	23 (88.5)	10 (90.9)	13 (86.6)
Ibuprofen, *n*(%)	11 (42.3)	6 (54.5)	5 (33.3)
Inv. Vent. (days)	22.9	28.2	19.1
NIV (days)	39.2	36.9	40.9
Antenatal steroids, *n*(%)	17 (65.4)	9 (81.8)	8 (53.3)
AKI, *n*(%)	8 (30.7)	6 (54.5)	2 (13.3)
IVH (grade III-IV), *n*(%)	5 (19.2)	3 (27.3)	2 (13.3)
PDA, *n*(%)	17 (65.4)	8 (72.7)	9 (60)
PHT (on sildenafil), *n*(%)	2 (7.7)	2 (18.2)	0
NICU stay (days)	120.7	121.8	119.9

The percentages for therapy and comorbidities refer to the patient group in the same column. *GA*, gestational age; *HTN*, hypertension; *BPD*, bronchopulmonary dysplasia; *AKI*, acute kidney injury; *IVH*, intraventricular haemorrhage; *PDA*, persistent ductus arteriosus; *PHT*, pulmonary hypertension; *Inv*. vent., invasive ventilation; *NIV*, noninvasive ventilation; *n*, number

### Measurement numbers

Covering the first 90 days of life, our database contained 19,481 measured blood pressure data separately for systolic blood pressure (SBP), diastolic blood pressure (DBP), and mean blood pressure (MBP). Therefore, the average number of raw data points per patient was 749.3 for each blood pressure category. This amounted to an average of 8.3 daily blood pressure measurements per patient. For the investigated period, the median of the measurement numbers was 180 (IQR 318.5–125).

### The evolution of the average systolic, diastolic and mean blood pressures in the BPD patient group compared to the reference blood pressure data

**Fig. 1:**
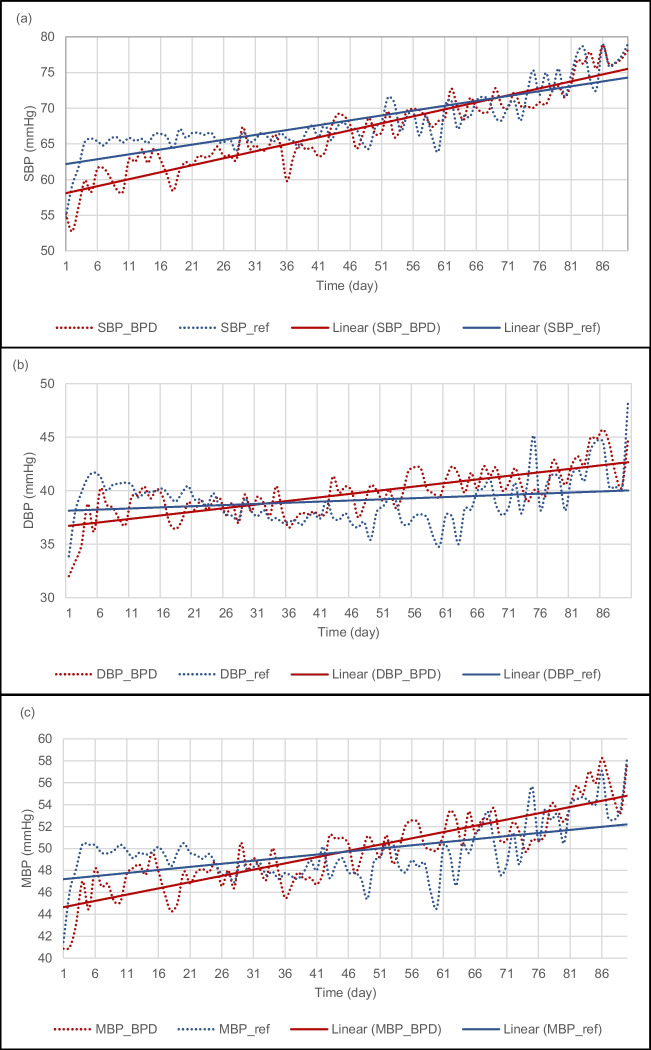
Systolic (**a**), diastolic (**b**) and mean (**c**) blood pressure curves and linear trendlines of the BPD group and the reference group as a function of time. *SBP*, systolic blood pressure; *BPD*, bronchopulmonary dysplasia; *ref*, reference group; *DBP*, diastolic blood pressure

We calculated the daily average systolic, diastolic and mean blood pressure values of the BPD patient group. Based on the daily average blood pressure data, we generated two trendlines: one for the BPD group and the other for the reference data. We compared the blood pressure curve and the corresponding trendline to the reference group's average daily blood pressure. The BPD patients' SBP trendline initially had lower values and rose gradually until around the 70th day of life, when it crossed the reference trendline (see Fig. 1a). The DBP and the MBP showed similar trends to that of the SBP, but they reached the reference trendline around the 30th and the 45th days of life, respectively (see Figs. 1b and c). We performed a separate statistical analysis for the time intervals before and after the intersection of the trendlines. The calculations revealed a significant correlation between the BPD-related data and the reference data (SBP: R1 = 0.732, R2 = 0.754, *p* < 0.001; DBP: R1 = 0.454, R2 = 0.625, *p *< 0.005; MBP: R1 = 0.371, R2 = 0.699, *p* < 0.012). However, there was a significant difference between the paired SBP, DBP and MBP curves (the calculated averages of the differences were as follows: SBP: M1 = -1.681, *p* < 0.001, M2 = -0.542, *p* = 0.139; DBP: M1 = -1.235, M2 = 1.627, *p* < 0.001; and MBP: M1 = -1.454, M2 = 1.502, *p* < 0.001).

### Comparison of the daily average blood pressure to the 95th-percentile blood pressure trendline

We calculated and compared each patient's daily average blood pressure to the 95th-percentile trendline. The average number of daily blood pressure measurements per patient was 8.3. At least three daily measurements were taken for all patients, ensuring that a single higher measurement was not used to diagnose hypertension. The average daily measurements per patient during the hypertensive episodes were 9.2 and 9.4 for the SBP and DBP, respectively.

Considering the 2340 patient days, we found 38 systolic (1.6%) and 50 diastolic (2.1%) daily average measurements above the 95th percentile. In our whole sample, only nine patients had no daily average blood pressure spikes (i.e., a systolic or diastolic daily average blood pressure above the 95th percentile), and 65% (n = 17) had at least one day with a hypertensive average daily blood pressure in the first three months of life. We found 13 patients (50%) who had average daily SBP and 16 patients (61.5%) who had average daily DBP values that exceeded the 95th percentile. Most of them had occasional daily blood pressure spikes. Eleven patients (42%) had three or more days with an average blood pressure above the 95th percentile.

We compared the patients with elevated blood pressure values for three or more days to the remaining patients (see Table 1). Our detailed statistical analysis showed that patients with three or more hypertensive days had a 25% chance of having AKI (H_0_: TP = 0.25, exact sig. = 0.034). AKI episodes (91.6%) mainly occurred within the first two weeks of life. All the investigated BPD patients had normal kidney function (i.e. age-appropriate serum creatinine levels and urine output) after the fifth week of life.

**Fig. 2:**
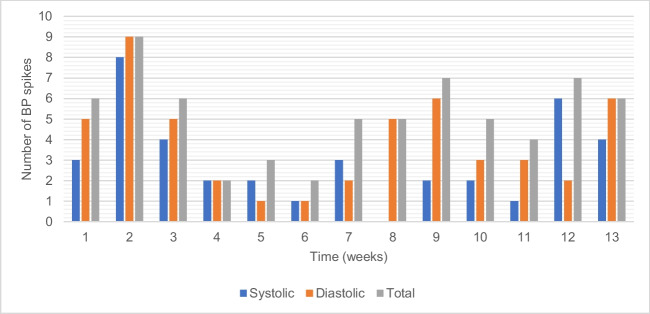
Number of systolic, diastolic, and total blood pressure spikes over 13 weeks. *BP*, blood pressure; *Total*, either systolic or diastolic BP spikes

We also examined the onset of the blood pressure increase. Most daily average hypertensive blood pressure measurements occurred at the 2nd, 9th, 12th, and 13th weeks of life, corresponding to the 28th, 35th, 38th, and 39th week of corrected gestational age, respectively (see Fig. 2). We found that 34%, 22%, and 44% of the hypertensive daily average blood pressures occurred in the first, second, and third months of life, respectively.

We investigated fluid bolus, blood transfusion and inotropic data in the database and found that the duration of administration did not coincide with periods of high blood pressure. Inotropic support was administered to 73% of the patients. All patients received blood transfusions, and the average transfusion number was 7.6 per patient for 90 days. A fluid bolus was administered to 16 patients. The average number of treatments administered was 1.75 per patient. These therapies were given mainly within the first three weeks of life: 44% of blood transfusions, 88% of fluid boluses and 62% of inotropes. The use of these therapies was limited compared to the entire investigated time interval. Therefore, these factors should not influence the results of our blood pressure analysis.

### Comparison of the weekly average blood pressure to the 95th-percentile blood pressure trendline

We calculated the weekly average blood pressure values for every BPD patient. The weekly average SBP and DBP values were above the 95th percentile in 3 (11.5%) of the 26 patients for one week only. These results indicate that some of the shorter hypertensive periods may remain hidden when weekly averages or averages from longer time intervals are used to obtain blood pressure measurements.

## Discussion

Our study aimed to examine blood pressure changes in BPD patients during the first three months of life. The length of the investigated period and the frequency of data collection, together with the high number of blood pressure measurements, make our study different from other related studies. The results of our detailed analysis using average daily blood pressure values show that the blood pressure values of BPD patients start lower and end higher than the reference value. To explain this phenomenon, we examined individual blood pressure changes in BPD patients with attention given to the influencing therapies. The individual daily average blood pressure calculations highlighted short periods with elevated blood pressure values. These blood pressure variations might partly explain the general blood pressure characteristics of the BPD patient group. We examined several factors influencing blood pressure, and our detailed statistical analysis showed that patients with three or more hypertensive days had a 25% chance of having AKI.

Several clinical studies have confirmed the increased risk of hypertension in BPD patients [3–8], while other studies have not shown a risk of high blood pressure in premature infants with BPD [11, 12]. In addition to the different diagnostic criteria used for BPD and hypertension, this difference could be due to the various and short periods during which blood pressure measurements were taken. The most likely onset time of hypertension varies widely, from 0.5 to 15 months of life [3, 4, 6]. Jenkins and colleagues reported that hypertension began at a mean age of 11.3 ± 3.2 chronological weeks and that the mean PMA was 39.6 ± 3.6 weeks [5]. In our patient group, the likelihood of a blood pressure increase was high in the 2nd, 9th, and 12th to 13th weeks of life. The latter corresponds to 38–39 weeks of corrected gestational age. An early increase in blood pressure might be related to other pathologies, such as AKI. However, a blood pressure increase beginning in the third month of life can be associated with BPD.

Our study has some limitations that arise from its retrospective nature. Since our data were retrospective, we could only draw limited conclusions on the effect of medications and therapy on blood pressure. An earlier study found a significant increase in blood pressure during dexamethasone therapy, which remained above baseline after treatment [25]. Based on the findings of a recent meta-analysis, a medium cumulative dose of postnatal dexamethasone is associated with a risk of hypertension [26]. On the other hand, several studies investigating hypertension in BPD patients did not demonstrate a hypertensive effect of postnatal steroids on the studied patient groups [4, 6, 7]. Most of our BPD patients received postnatal steroids, which might have affected their blood pressure. Since 88.5% of the patients received diuretics due to BPD, this may have influenced the number and length of hypertensive episodes. Previous research on BPD-related blood pressure changes has not detailed the effects of diuretic therapy on the development of blood pressure changes. There are several general therapies (e.g., blood transfusions, fluid boluses, inotropic support) used during the treatment of preterm infants with BPD, which might cause changes in blood pressure. An extensive and prospective study would be useful to further clarify the effect of these influencing factors. Another limitation of our research is that we had to exclude patients from the study who died since it was not possible to determine their BPD status.

The aetiology of hypertension in BPD patients is still unknown. One of the most studied explanations is that the systemic effects of kidney failure negatively affect the developing lungs [27, 28]. Animal studies have shown that AKI affects angiogenesis and alveolarisation, leading to a BPD phenotype in experimental animals [29]. In our study, the incidence of AKI was significantly greater among patients with three or more hypertensive days. Some studies have investigated the association between pulmonary and systemic hypertension, but no correlation has been verified [6].

To investigate the relationship between AKI, BPD, hypertension, and cardiac function, a larger, prospective study is needed. Collecting blood pressure follow-up data from the BPD patient group and investigating daily blood pressure changes in AKI could improve our understanding of blood pressure changes in preterm infants with different underlying disorders.

## Conclusion

The blood pressure of the BPD patient group was not only correlated but also significantly different from that of the reference data. By examining the daily average blood pressure, it is possible to diagnose hypertensive episodes that cannot be detected by calculating weekly averages or by analysing shorter time periods than three months. The recognition of hypertensive periods allows for early and appropriate treatment and helps clinicians determine which patients need regular blood pressure follow-ups in infancy. Hypertensive blood pressure values for three or more days occurred more frequently in patients with AKI accompanied by BPD. However, further studies are needed to determine the influencing factors and pathophysiology of hypertension in BPD patients.

### Supplementary Information

Below is the link to the electronic supplementary material.

Graphical abstract (PPTX 169 KB)

## Data Availability

Data are available upon request from the authors.
