# Supplementary material for: Blood pressure in preterm infants with bronchopulmonary dysplasia in the first three months of life
Source: Pediatr Nephrol. 2024 Mar 27;39(8):2475–81. doi: 10.1007/s00467-024-06354-0 (PMC11199223; doi:10.1007/s00467-024-06354-0)
Supplement: Supplementary file 1 — Graphical abstract (PPTX 169 KB) [file 467_2024_6354_MOESM1_ESM.pptx]

## Slide 1
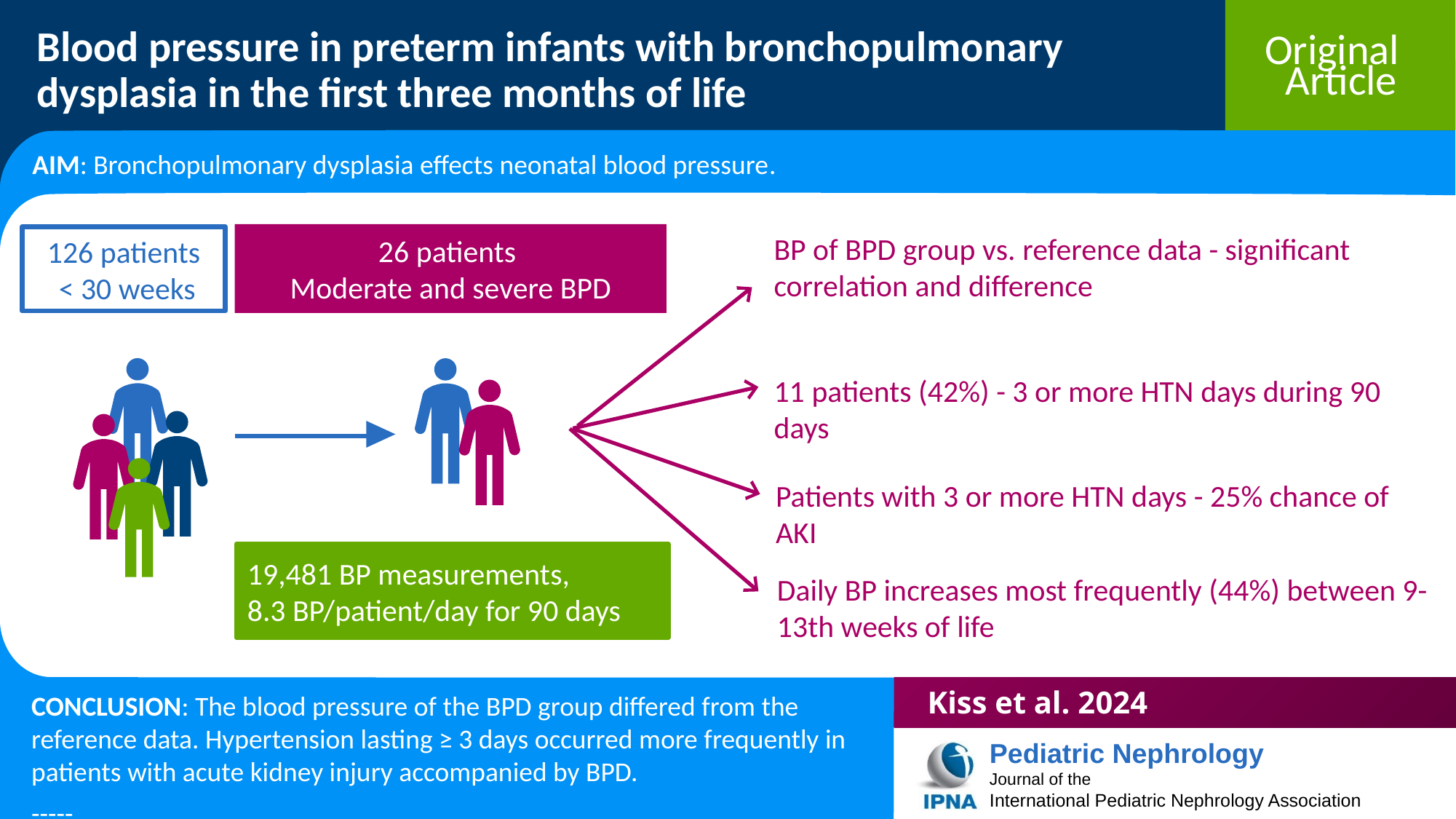

Blood pressure in preterm infants with bronchopulmonary dysplasia in the first three months of life
AIM: Bronchopulmonary dysplasia effects neonatal blood pressure.
26 patients
Moderate and severe BPD
BP of BPD group vs. reference data - significant correlation and difference
126 patients
 < 30 weeks
11 patients (42%) - 3 or more HTN days during 90 days
Patients with 3 or more HTN days - 25% chance of AKI
19,481 BP measurements,
8.3 BP/patient/day for 90 days
Daily BP increases most frequently (44%) between 9-13th weeks of life
Kiss et al. 2024
CONCLUSION: The blood pressure of the BPD group differed from the reference data. Hypertension lasting ≥ 3 days occurred more frequently in patients with acute kidney injury accompanied by BPD.
-----
